# Dissecting Gonadoblastoma of the Ovary Coexistent with an Atypical Endometriotic Cyst: Incidental Detection in Cystectomy Specimen of a Woman with 46,XX Karyotype

**DOI:** 10.3390/diagnostics12030660

**Published:** 2022-03-09

**Authors:** Hera Jung, Bo Seong Yun, Yoon Yang Jung, Hyun-Soo Kim

**Affiliations:** 1 Department of Pathology, CHA Ilsan Medical Center, CHA University School of Medicine, Goyang 10414, Korea; elledriver2008@gmail.com; 2 Department of Obstetrics and Gynecology, CHA Ilsan Medical Center, CHA University School of Medicine, Goyang 10414, Korea; boseong.yun@gmail.com; 3 Department of Pathology, Myongji Hospital, Hanyang University College of Medicine, Goyang 10475, Korea; 4 Department of Pathology and Translational Genomics, Samsung Medical Center, Sungkyunkwan University School of Medicine, Seoul 06351, Korea

**Keywords:** ovary, dissecting gonadoblastoma, atypical endometriotic cyst, normal karyotype

## Abstract

Dissecting gonadoblastoma (DGB) of the ovary, a recently described terminology, defines a unique distribution of neoplastic germ cells. Here, we report a case of incidental DGB coexistent with an atypical endometriotic cyst occurring in a 23-year-old woman. The ovarian cyst was lined by endometrial-like glands and stroma. Some glands displayed nuclear enlargement and hyperchromasia, and abundant eosinophilic cytoplasm with occasional intracytoplasmic hemosiderin and mucin vacuoles. The neoplastic germ cells resembled those of ovarian dysgerminoma and were diffusely distributed within the ovarian stroma, which was stretched around the wall of the endometriotic cyst. These cells were arranged in nests and cords, possessing clear cytoplasm and centrally located round nuclei with prominent nucleoli and occasional mitoses. Chromosomal analysis revealed a 46,XX karyotype. We describe the clinical, histological, immunophenotypical, and genetic features of ovarian DGB incidentally detected in the ovarian cystectomy specimen of a woman with normal female karyotype.

Gonadoblastoma is a rare gonadal neoplasm consisting of a mixture of steroid hormone-producing germ cells, sex cord cells, and stromal cells [1]. Scully [2] reviewed 74 cases of ovarian gonadoblastoma and reported sexual abnormalities in each patient. Since then, several case series and individual case reports of ovarian gonadoblastoma harboring normal karyotype have been reported in the literature [3,4,5,6,7,8,9,10,11,12,13,14,15,16]. These patients did not have any sexual developmental disorder and most were females with a 46,XX karyotype. Recently, Kao et al. [17] described a “dissecting gonadoblastoma (DGB)”, which is a morphological variant of gonadoblastoma. Their research documented 11% of patients with ovarian gonadoblastoma possessing 46,XX karyotype, and that, histologically, DGB was typically accompanied by areas of classic gonadoblastoma [17]. DGB is a variant of classical gonadoblastoma, exhibiting unusual growth patterns and containing germ cell and sex cord elements [18]. We recently experienced a rare case of ovarian DGB in a 23-year-old woman with 46,XX karyotype. The patient underwent a cystectomy for an ovarian endometriotic cyst where the DGB tissue was incidentally found during microscopic examination of the cystectomy specimen. The 23-year-old woman presented with intermittent low abdominal pain. She visited a local emergency room three years prior because of severe dysmenorrhea. At that time, a left ovarian mass was first detected via transvaginal ultrasonography (US). Oral contraceptives were administered for three months, and the mass size decreased. However, she visited the local clinic again in response to low abdominal pain that lasted for six months. She was referred to our institution with the possibility of an ovarian tumor. A preoperative US revealed a 6.5 cm × 6.1 cm hypoechoic cystic mass in the left adnexa (Figure 1), suggestive of an ovarian endometriotic cyst. Serum levels of cancer antigen (CA) 125 were elevated to 53.8 U/mL, whereas the levels of serum CA 19-9, CA 15-3, carcinoembryonic antigen, and α-fetoprotein were within normal ranges. She underwent a laparoscopic cystectomy. Based on the histological features (Figure 2) and immunostaining results (Figure 3), the diagnosis of ovarian DGB coexistent with an atypical endometriotic cyst was established. Chromosomal analysis on a peripheral blood specimen revealed 46,XX normal female karyotype (Figure 4). Targeted next-generation sequencing analysis revealed no pathogenic mutation in genes that are implicated in homologous recombinant repair, excluding the possibility of hereditary breast and ovarian cancer syndrome. The patient reported a normal menstrual cycle and no history of sexual developmental abnormality. At three months after surgery, an abdominopelvic computed tomography revealed no evidence of recurrent disease. The serum level of CA 125 was within the normal range. However, at eleven months postoperatively, the serum CA 125 level was slightly elevated to 37.9 U/mL and a follow-up US revealed a newly identified left ovarian cyst. Oral progestin was administered based on the clinical impression of recurrent ovarian endometriotic cyst. Serum CA 125 levels decreased to 15.2 U/mL after three months of medication.

The differential diagnosis of DGB includes classic gonadoblastoma, dysgerminoma, and undifferentiated gonadal tissue (UGT). Classic gonadoblastoma consists of primitive germ cells, sex cord cells, and stromal cells. They are typically arranged in multiple, round-to-ovoid nests surrounded by hyalinized basement membrane. Microcalcifications can be seen in the center of these nests. Meanwhile, DGB has the same cellular components as those of classic gonadoblastoma; however, patterns of cellular growth are different from classic type. DGB usually exhibits three growth patterns, including solid/expansile, anastomosing, and cord-like. Even though solid/expansile-type DGB can be confused with dysgerminoma, the latter shows no sex cord–stromal component. The presence of sex cord cells and the absence of granulomatous reaction can aid pathologists in distinguishing DGB from dysgerminoma [17]. Anastomosing and cord-like types of DGB are characterized by small, inter-anastomosing trabeculae, nests, and cords. Since the sex cord cells are more subtle than classic type, inhibin-α immunostaining can be useful to highlight these sparsely distributed sex cord cells. UGT also differs from DGB. This non-neoplastic, non-mass forming condition is not as prominent as DGB and exhibits a predominant gonadal stromal component. UGT blends within gonadal stroma and is not easily discriminated from the background. In contrast, DGB forms a distinct mass and more readily presents with germ cell and sex cord elements. Although gonadoblastoma primarily arises from dysgenetic gonads, exceptional cases of gonadoblastoma have been reported in women with a normal karyotype. Kim et al. [8] reported two phenotypically normal women with 46,XX karyotype who were diagnosed with gonadoblastoma with cord-like growth patterns. One of the two patients possessed an ovarian endometriotic cyst combined with gonadoblastoma. Susman et al. [10] also described a patient with 46,XX karyotype who had bilateral ovarian gonadoblastomas coexistent with endometriotic cysts.

In summary, we observed some unique clinicopathological features: (1) incidental gonadoblastoma showing a dissecting morphology; (2) coexistence with ovarian atypical endometriotic cyst; and (3) a cytogenetically and phenotypically normal female. Our observations allow pathologists to recognize and accurately diagnose this rare entity.

## Figures and Tables

**Figure 1:**
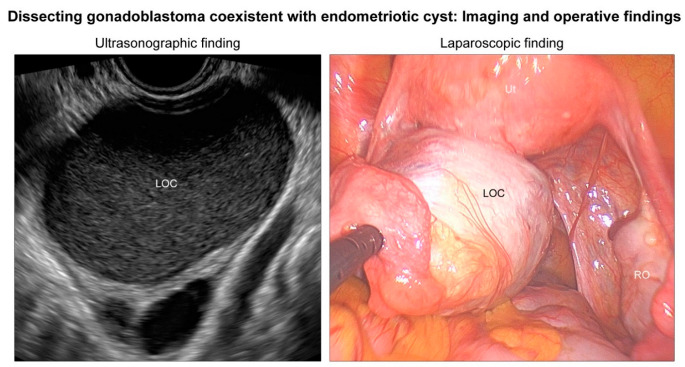
Imaging and operative findings. A 23-year-old woman presenting with intermittent low abdominal pain was referred to our institution with the possibility of an ovarian tumor. Preoperative ultrasonography revealed a 6.5 cm hypoechoic cystic mass in the left adnexa (LOC), suggestive of an ovarian endometriotic cyst. She underwent a laparoscopic left ovarian cystectomy. During laparoscopy, an endometrioma-like cystic mass was detected. The LOC contained chocolate-like fluid. The right ovary (RO) and uterus (Ut) were unremarkable except for a focal filmy adhesion between the anterior uterine serosa and peritoneum. No cul-de-sac obliteration was identified.

**Figure 2:**
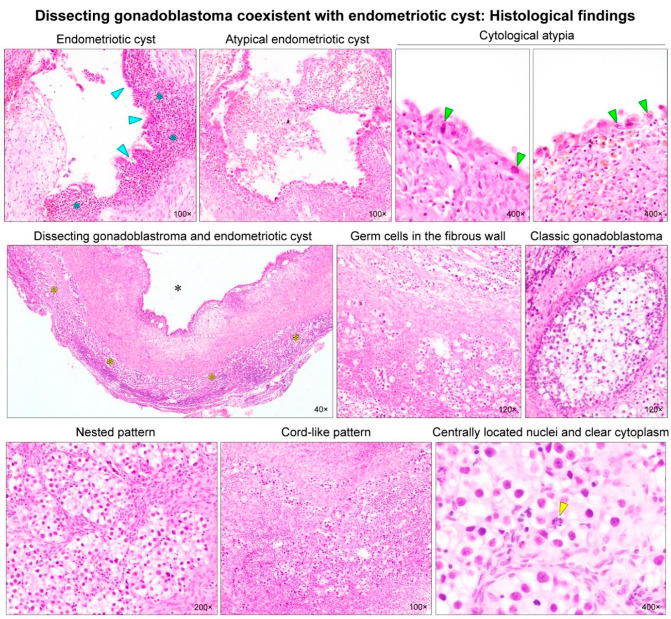
Histological findings. The left ovarian cyst measured 4.8 cm at its greatest dimension, with a smooth and glistening external surface. Serial sections revealed an irregularly thickened unilocular cystic wall and chocolate-like fluid. No solid component or necrosis was identified. Histologically, the inner surface of the cyst was lined by endometrial-like glands (blue arrowheads) and stroma (blue asterisks). In some foci, the glandular epithelial cells showed variable degrees of cytological atypia (green arrowheads), including nuclear enlargement and hyperchromasia, abundant eosinophilic cytoplasm with occasional intracytoplasmic hemosiderin and mucin vacuoles, and low nuclear-to-cytoplasmic ratio. Architectural distortion or confluent glandular crowding was absent. These histological features were compatible with an atypical endometriotic cyst. In addition, numerous germ cells resembling those of ovarian dysgerminoma were diffusely distributed within the ovarian stroma (yellow asterisks), which was stretched around the wall of the endometriotic cyst (black asterisk). A few foci of classic gonadoblastoma were seen. Although most of the neoplastic germ cells formed small nests, some were arranged in cords or trabeculae. Under high-power magnification, the cells possessed clear cytoplasm and were centrally located around the nuclei with occasional prominent nucleoli. Mitotic figures (yellow arrowhead) were readily identifiable.

**Figure 3:**
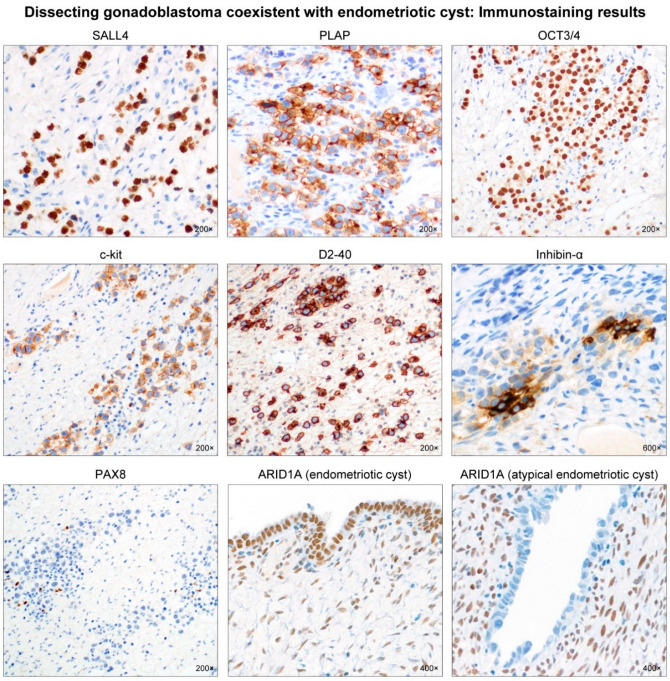
Immunostaining results. The neoplastic germ cells were positive for spalt-like protein 4 (SALL4), placental alkaline phosphatase (PLAP), octamer-binding transcription factor 3/4 (OCT3/4), c-kit, and D2-40. Some sex cord cells between germ cell nests expressed inhibin-α. The neoplastic germ cells were negative for paired box 8 (PAX8), a Mullerian-lineage epithelial marker. AT-rich interaction domain 1A (ARID1A) expression was uniformly positive in the glandular epithelium of typical endometriotic cyst, whereas ARID1A immunoreactivity was absent in the lining epithelium of atypical endometriotic cyst.

**Figure 4:**
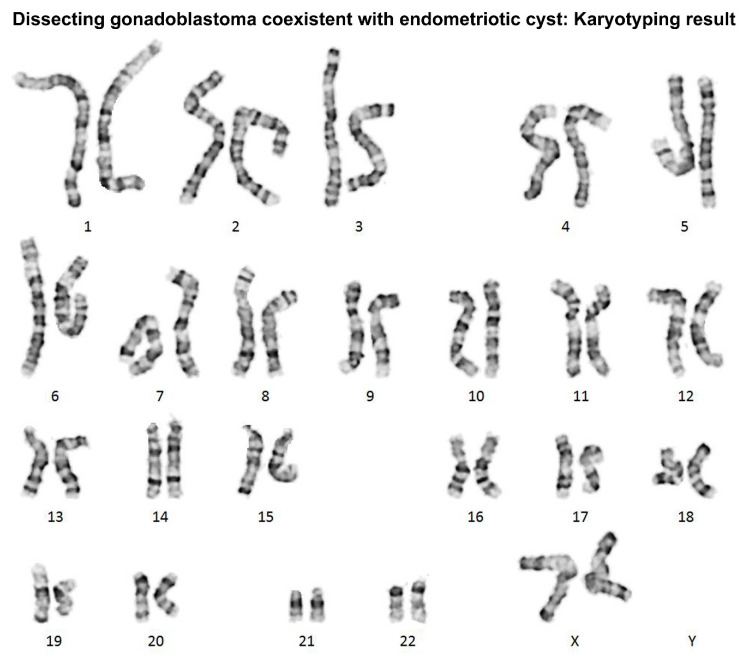
G-banding karyotyping result showing the pictorial alignment of the 22 pairs of homologous autosomes from one metaphase cell, sequentially numbered from chromosome 1 to 22 by their unique band patterns, and the two sex chromosomes, XX (female) or XY (male). The patient has a 46,XX normal female karyotype.

## Data Availability

Not applicable.
